# High-dose daptomycin and fosfomycin treatment of a patient with endocarditis caused by daptomycin-nonsusceptible *Staphylococcus aureus*: Case report

**DOI:** 10.1186/1471-2334-11-152

**Published:** 2011-05-26

**Authors:** Liang-Yu Chen, Cheng-Hsiung Huang, Shu-Chen Kuo, Chen-Yuan Hsiao, Mei-Lin Lin, Fu-Der Wang, Chang-Phone Fung

**Affiliations:** 1 Division of Infectious Diseases, Department of Medicine, Taipei Veterans General Hospital, Taipei, Taiwan; 2 Division of Cardiovascular Surgery, Department of Surgery, Taipei Veterans General Hospital, Taipei, Taiwan; 3 School of Medicine, National Yang-Ming University, Taipei, Taiwan

**Keywords:** Daptomycin-nonsusceptible, fosfomycin, ICD device-related endocarditis

## Abstract

**Background:**

Emergence of daptomycin-nonsusceptible (DNS) *Staphylococcus aureus *is a dreadful problem in the treatment of endocarditis. Few current therapeutic agents are effective for treating infections caused by DNS *S. aureus*.

**Case presentation:**

We describe the emergence of DNS *S. aureus*. in a patient with implantable cardioverter-defibrillator (ICD) device -related endocarditis who was priorily treated with daptomycin. Metastatic dissemination as osteomyelitis further complicated the management of endocarditis. The dilemma was successfully managed by surgical removal of the ICD device and combination antimicrobial therapy with high-dose daptomycin and fosfomycin.

**Conclusions:**

Surgical removal of intracardiac devices remains an important adjunctive measure in the treatment of endocarditis. Our case suggests that combination therapy is more favorable than single-agent therapy for infections caused by DNS *S. aureus*.

## Background

Infective endocarditis is a clinically significant disease with mortality ranging from 16% to 25% [1,2], and *Staphylococcus aureus *is reported as the most common pathogen, accounting for 31.4% of cases in developing countries [3]. In addition to methicillin-resistant *S. aureus *(MRSA), the emergence of vancomycin-resistant *S. aureus *also became a major problem after the first report in Japan in 1997 [4].

Daptomycin is a 13-aminoacid compound derived from fermentation of *Streptomyces roseosporus *and is a new treatment option for *S. aureus *endocarditis, including vancomycin-resistant *S. aureus *[5,6]. The once daily dosing of daptomycin 6 mg/kg approved by the USA Food and Drug administration showed non-inferiority to the standard therapy for treating *S. aureus *bacteremia and endocarditis [7]. However, increased incidence of daptomycin-nonsusceptible (DNS) *S. aureus *was reported recently. Mutations in the *mprF *and *yycFG *genes, related to membrane fluidity changes or fatty acid biosynthesis, were identified recently as possible contributors to daptomycin nonsusceptibility [5,8]. Effective management in case of DNS *S. aureus *infections is an important issue nowadays.

Fosfomycin, a phosphonic acid derivative (cis-1,2-epoxypropyl phosphonic acid), was initially described and isolated in 1969 from cultures of *Streptomyces *species [9]. It was commonly employed in treating urinary tract infections and has a bactericidal mechanism of action [9,10]. Because it is a relatively small, hydrophilic molecule with almost negligible serum protein binding and good tissue penetration, this agent was more frequently used in combination therapy for treating multidrug-resistant pathogens [10,11].

We present our treatment experience utilizing surgical intervention and high-dose daptomycin in combination with fosfomycin for successful treatment of a patient with implantable cardioverter-defibrillator (ICD) device-related endocarditis complicated with osteomyelitis caused by DNS MRSA.

## Case Presentation

A 28-year-old woman had a history of sudden onset of syncope diagnosed as paroxysmal ventricular tachycardia. She underwent electric ablation and placement of an ICD in her left shoulder. She had poor wound healing at a 5-month follow-up visit and she was admitted to the hospital on December 1, 2008 for management of a pocket infection. Exposure of the ICD generator and pacing lead were found on February 26, 2009, and the generator was removed, leaving the pacing lead in place. She had episodic pyrexia beginning in May 2009 and her blood cultures grew MRSA (isolate st01, positive in one of two sets blood cultures collected). ICD device-related endocarditis was highly suspected. Intravenous antibiotics with vancomycin 1 g every 12 hours for 14 days followed by oral linezolid 600 mg every 12 hours for another 14 days were prescribed for her endocarditis, but the blood cultures still grew MRSA (isolate st02, positive in one of two sets blood cultures collected). A thoracotomy was planned to remove the pacing lead. However, because of three episodes of broken peripheral venous catheter tips left in the vessels, generator displacement requiring surgical intervention, and emotional fragility, the surgical intervention was postponed due to the high possibility of postoperative wound infection and further osteomyelitis. Daptomycin at a dosage of 6 mg/kg daily was prescribed for its effective biofilm penetration ability and rapid bacteriocidal efficacy, and the subsequent blood culture (one set collected) yielded negative finding. A total 65 days of treatment were completed and the patient was discharged on October 24, 2009.

Unfortunately, spiking fever and chills occurred 1 day after discharge and the blood cultures grew MRSA again (isolates st03, positive in one of two blood cultures collected). During antibiotic treatment with intravenous tigecycline (100-mg loading dose and 50 mg every 12 hours for 14 days), one of two sets of blood cultures grew MRSA (isolate st04). Teicoplanin 400 mg every 12 hours for another 14 days was given, but another one of two sets of blood cultures grew MRSA (isolate st05). Intravenous daptomycin was prescribed at a dosage of up to 9 mg/kg daily in combination with intravenous fosfomycin 6 g every 6 hours starting December 16, 2009, due to the persistence of MRSA bacteremia with the ICD device in place. The patient's fever subsided after these treatments and the next three sets of blood cultures were negative 1 week after the start of daptomycin-fosfomycin combination therapy. However, transesophageal echocardiography revealed vegetations growing on the pacing lead, and a scan for osteomyelitis showed increased uptake of the radiotracer in the left proximal clavicle. Moreover, the daptomycin Etest (AB Biodisk, Solna, Sweden) of MRSA isolate st05 indicated that it was nonsusceptible to daptomycin, with a minimal inhibitory concentration (MIC) ranging from 1.5 to 2 mg/L. After discussion among the surgeons and infectious disease specialists, a thoracotomy was performed on December 22, 2009 for removal of the pacing lead due to the previous persistence of *S. aureus *bacteremia; high-dose daptomycin 12 mg/kg intravenously daily was also started. A vegetation measuring 1.3 × 0.6 × 0.1 cm was found on the pacing lead, but the Gram stain showed no microbes. Tissue culture of the vegetation found on the pacing lead grew MRSA (isolate st06), as did as the tip of the pacing lead (isolate st07).

Because the osteomyelitis scan was positive, combination therapy with daptomycin 12 mg/kg intravenously daily and fosfomycin 6 g intravenously every 6 hours was administered for total of 56 days. The patient had no further pyretic episodes following hospitalization. A follow-up osteomyelitis scan on March 8, 2010 was negative, and follow-up transthoracic echocardiography showed no vegetation. The patient's symptoms were attributed to endocarditis; she remained symptom free during 12 months of follow-up.

## Methods

The species identification was completed using the API 32GN biochemical testing system (bioMérieux, Inc., Marcy l'Étoile, France). Antimicrobial susceptibility testing was performed using agar dilution method with 25 mg/L glucose-6 phosphate added for fosfomycin testing; the broth dilution method of antimicrobial susceptibility testing was used for the other antimicrobial agents, as specified by the Clinical Laboratory Standards Institute (CLSI 2010), using the VITEK^® ^2 Compact system (bioMérieux, Inc.,) and daptomycin Etest strips (AB Biodisk, Solna, Sweden). Guidance for interpreting the MICs as clinical categories (susceptible, intermediate, or resistant) was provided by the manufacturer's instructions and CLSI criteria. Isolates with daptomycin MICs ≤ 1 mg/L were defined as daptomycin-susceptible, and those with MICs > 1 mg/L were defined as daptomycin-nonsusceptible. No MIC interpretation criteria were available for fosfomycin on bacteremia in CLSI criteria, but isolates with MICs less than 32 mg/L were defined as susceptible by the European Committee on Antimicrobial Susceptibility Testing (EUCAST). In order to establish the clonality of these isolates, we performed pulsed-field gel electrophoresis (PFGE) as described previously [12]. *S. aureus *ATCC 29213 was used as the quality control organism.

## Results

The seven isolates were analyzed by their Sma1 PFGE profiles and isolates st02 to st06 showed the same profile, while isolates st01 and st07 were different. Progressive elevation of the daptomycin MICs for isolates of the same clonality occurred, but decreasing MICs for isolate st06 were noted for vancomycin, teicoplanin, and daptomycin compared to those of isolate st05 (Table 1). The MICs of fosfomycin for all isolates were less than 2 mg/L by the agar dilution methods and showed susceptibility based on the EUCAST criteria. All isolates were resistant to the other antimicrobials screened using the VITEK^® ^2 Compact system (Table 1).

**Table 1:** Antimicrobial susceptibility results for the seven methicillin-resistant *Staphylococcus aureus *clinical isolates

Isolate	Date of isolation	MIC (mg/L)							
		**Vitek 2**			**Broth dilution**			**Agar dilution**	**Etest**

		**VAN**	**TEIC**	**LIN**	**VAN**	**TEIC**	**DPC**	**FOS**	**DPC**

st01	2009/5/19	< 0.5	< 0.5	2	1	1	0.25	< 2	0.19
st02	2009/9/27	2	< 0.5	2	1	1	0.25	< 2	0.38
st03	2009/10/29	2	< 0.5	4	1	1	0.5	< 2	0.25
st04	2009/11/10	1	< 0.5	4	1	1	0.5	< 2	0.25
st05	2009/12/11	2	2	4	2	0.25	1	< 2	1.5-2
st06	2009/12/22	2	< 0.5	2	1	0.5	0.5	< 2	0.25
st07	2009/12/22	2	1	4	2	4	2	< 2	1

DPC: daptomycin; FOS: fosfomycin; LIN: linezolid; MIC: minimal inhibitory concentration; TEIC: teicoplanin; VAN: vancomycin

## Conclusions

Combination therapy consisting of high-dose daptomycin and fosfomycin is an alternative treatment option for DNS MRSA native valve endocarditis or osteomyelitis. These two agents are well known for their bacteriocidal activities and effective biofilm penetration [5,6,10,11]. Recently, positive results for fosfomycin used in combination with other antimicrobial agents against multidrug resistant Gram-positive pathogens were also reported [9,11].

Isolate st06 from the vegetation, of the same clonality as isolate st05, was susceptible to vancomycin, teicoplanin, and daptomycin in combination with fosfomycin with additive effect. Additionally, the pathological findings of the vegetation were negative for microbes, which suggested that the combination regimen was highly effective in biofilm penetration and killing the bacteria. Moreover, the osteomyelitis was successfully treated in 8 weeks, without complications. Although high-dose daptomycin treatment could be effective after surgical removal of an ICD-device for adequate pharmacodynamics attainments, the combination therapy is preferred in complicated cases, and further study to establish the adequate combination dosage of daptomycin with fosfomycin is needed. Steed et al. also reported that the combination of daptomycin plus clotrimoxazole was bactericidal against DNS MRSA in an in vitro model of simulated endocardial vegetations [13].

High-dose daptomycin treatment (12 mg/kg daily) was well tolerated in our patient throughout the 8-week treatment course. In a previous study, patients received daptomycin in dosages greater than 8 mg/kg per day, and 79% of patients were considered evaluable for efficacy; only 6.4% experienced 1 or more adverse events or abnormal laboratory analyte values [14]. Another recent report also revealed no remarkable creatinine kinase elevation after daptomycin use in dosage greater than 8 mg/kg once daily for a prolonged course of 14 days [15]. Because the resistance mechanism of *S. aureus *to daptomycin is unique in comparison to other glycopeptides, and every single gene mutation, such as *mprF *and *yycFG *genes, causes only a slight increase in daptomycin MIC, higher doses of daptomycin could still be effective for the treatment of DNS *S. aureus *[5,8]. Nonetheless, in vitro and in vivo evidence suggests that combination therapy is more favorable than single-agent therapy for infections caused by DNS *S. aureus*, and antibiotic therapy must be individualized for each patient's condition.

For patients with ICD device-related endocarditis, surgical removal of the device is strongly recommended when *S. aureus *bacteremia persists after antimicrobial therapy [16,17]. As reported by del Rio A et al. the prevalence of treatment failure is greater among patients receiving only antibiotics therapy compared to those treated with surgical removal of implanted device and antibiotics therapy [18]. Recurrence of MRSA bacteremia identified during our patient's second visit suggested treatment failure, even though the blood cultures were negative after the initial daptomycin treatment; such patients should undergo prompt surgical removal of the ICD device. It is possible that with daptomycin alone the patient could have been cured in the first attempt if the ICD lead had been surgical removed. Although the combination therapy achieved negative blood cultures, the tissue culture of the vegetation and pacing lead still grew MRSA, with isolate st07 showing increased daptomycin resistance. Recurrence would have remained possible without the removal of the pacing lead.

## Consent

Written informed consent was obtained from the patient for publication of this case report.

## Competing interests

The authors declare that they have no competing interests.

## Authors' contributions

LYC, SCK, and MLL completed the microbial cultures, agar and broth microdilutions for MICs and PFGE. CHH and CYH took responsibility of the patient on admission for wound debridement, thoracotomy, and postsurgical care. FDW and CPF suggested and decided which antibiotics were administered and suggested the plan of action based on the advanced image examinations. All authors read and approved the manuscript.

## Pre-publication history

The pre-publication history for this paper can be accessed here:

http://www.biomedcentral.com/1471-2334/11/152/prepub
